# Surgical reconstruction is a cost-efficient treatment option for isolated PCL injuries

**DOI:** 10.1007/s00167-017-4632-5

**Published:** 2017-07-14

**Authors:** Christian Owesen, Eline Aas, Asbjørn Årøen

**Affiliations:** 1 Ortopedisk Klinikk, Ahus, 1478 Nordbyhagen, Norway; 2 Departement of Health Economics and Health Management, University of Oslo, Postboks1089, 0318 Oslo, Norway

**Keywords:** Health economics, Knee, Posterior cruciate ligament, Knee ligament, Knee registries, Cost of treatment of PCL injury

## Abstract

**Purpose and hypothesis:**

The main purpose of the study is to put focus on the costs related to treating posterior cruciate ligament (PCL) injuries and the possible implications of chosen treatment strategy to the respective institutions and society.

**Methods:**

Costs of treating PCL injuries nonoperatively and for both single-bundle (SB) and double-bundle (DB) reconstruction were estimated. These costs were translated into equivalent quality-adjusted life years (QALY) given a threshold value of Euro (€) 70,000 per QALY. Expected gain in knee osteoarthritis outcome score (KOOS) quality of life (QoL) following surgery based on KOOS data from 112 patients was used as a basis for calculating the cost efficiency ratio.

**Results:**

The average calculated cost of nonoperative treatment was €3382. Incremental cost for SB PCLR was €8585 (154%) and another increment of €5220 (61%) for DB PCLR using numbers from a European hospital. This is equivalent to increments of 0.074 (SB) and another 0.075 (DB) QALYs given the €70,000 threshold. For DB to be as cost efficient as SB reconstruction, the incremental gain in KOOS QoL has to be at the same level as for SB reconstruction compared to nonoperative treatment.

**Conclusion:**

Though surgical reconstruction adds a substantial cost to nonoperative treatment alone, it can be considered cost-effective. Double-bundle reconstruction is less cost efficient than SB reconstruction, but should probably still be considered the treatment of choice for certain patient categories. Randomized controlled trials looking at outcome following nonoperative, SB and DB PCL reconstruction are needed. The clinical relevance of this is that surgical reconstruction of PCL injuries is a cost-efficient treatment alternative in patients with an isolated PCL injury. This finding should be taken into consideration when deciding on how to treat these injuries.

**Level of evidence:**

III.

## Introduction

Posterior cruciate ligament (PCL) injuries are occurring in increasing frequency, and studies report that injuries to the PCL account for as much as 17% of all knee injuries [10]. Treating these injuries is related to a cost to the society. A majority of such injuries can be treated nonoperatively with a satisfactory result [6, 12, 17]. As a consequence, most patients suffering an isolated PCL injury traditionally go through a regime of rigid rehabilitation. If the injury is acknowledged within the first few weeks, the nonoperative approach usually consists of a PCL brace and a series of training exercises [6, 8]. If a nonoperative approach is unsuccessful, surgical reconstruction of the PCL is the alternative. In recent years, there has been increased focus on PCL reconstruction (PCLR). The PCL consists of two functional bundles [8]. Traditionally single-bundle (SB) reconstruction has been the preferred method of reconstruction, but double-bundle (DB) reconstruction has also been reported since 1999 [4]. DB PCLR commonly includes the use of allografts instead of or in addition to the hamstring graft as well as extra time in the operating room, which adds costs to a SB reconstruction with an autograft. For DB reconstruction, the results have not been reported to being superior to those of SB reconstructions until 2010. Describing a new technique with the use of two allografts, Spiridonov et al. [19] reported excellent outcomes both when it comes to objectively measured stability and patient-reported outcomes. Later, biomechanical studies have shown superior results with the same DB PCLR technique compared to SB technique [7, 20]. To our knowledge, there are no studies that have looked at the costs of the different treatment algorithms for treating PCL injuries. With the cost related to surgery for both SB and DB reconstructions, a gain in outcome should be required to justify the procedure over nonoperative treatment alone. As patient-reported outcome measure, the knee injury and osteoarthritis outcome score (KOOS) is most commonly used in the Scandinavian countries. If there is an improvement in the KOOS, following treatment is therefore an important question. Other important issues include whether the patients are able to return to work following surgery and the duration of the sick leave, health care utilization, and in addition cost per health care gain, measured in KOOS QoL. In a longer perspective, it is interesting to evaluate whether reconstruction has an effect on the development of osteoarthritis (OA) and whether choosing DB over SB reconstruction leads to a decrease in revision surgery rates.

This study aims to enlighten the cost of both operative and nonoperative treatments of PCL injuries and explore the cost per expected gain measured in quality of life following surgery.

## Materials and methods

### KOOS

The health outcome was measured by the KOOS questionnaire; a self-administered knee function score consisting of 42 questions is divided into five different subscales: pain, other symptoms, activities of daily living (ADL), function in sport/recreation and knee-related quality of life (QoL). It was developed in the 1990s by Roos et al. [16]. The KOOS includes the WOMAC Osteoarthritis Index in its complete and original format and is a validated and reliable tool for measuring knee function in patients with osteoarthritis (OA) and for several types of knee injury including ACL injuries, meniscal injuries and cartilage injuries [15]. Each subscale ranges from 0 (worst) to 100 (best). It is usually considered that a difference of 8–10 points in a subscale is a clinically relevant effect. It is recommended to evaluate each subscale independently when considering outcome measures [15]. KOOS is responsive to change following nonsurgical and surgical interventions. KOOS QoL is the KOOS subscale usually most responsive following orthopaedic treatment. Expected gain in health outcome after operative treatment was based on improvement in KOOS QoL data reported in a previous study, where the average increment at 2 years was 26.6 points [11].

### Costs

Costs of treating PCL injuries nonoperatively and operatively for both SB and DB techniques were estimated (Table 1). For all patients, the costs included a dynamic PCL brace and a standard period of rehabilitation (6 months) with guidance of a certified physiotherapist. One brace per patient was estimated, as the same brace can be used post-operatively for the patients requiring PCL reconstruction (PCLR). The brace cost was that of either Jack brace (Albrecht GmbH) or the newer Rebound PCL brace (Össur Inc) at Norwegian market price. These are currently the only two available dynamic PCL braces. The two braces have comparable prices. Two training sessions per week over a period of 26 weeks with a physiotherapist was estimated as sufficient in addition to independent training. There is no clearly defined rehabilitation protocol in the literature for nonoperative treatment of PCL injuries. A period of 26 weeks was chosen following recommendations in recent publications [3, 5, 6, 13, 18] and from the common practice at the authors’ institution. This protocol can be considered as a general recommendation, although individual adjustments in duration and intensity may sometimes be necessary. The cost of the rehabilitation programme was calculated using guidelines provided by the Norwegian Physiotherapist Association, here estimated to €44.6 average cost per session. Sixty minutes was estimated for the first session and 30 min for the remaining sessions. The cost per hour and modality of treatment will vary according to the country or institution where the patient is treated.

**Table 1:** Resource use and estimated unit cost (in €) according to treatment strategy

Cost component	Units			Unit price	Source
	No^a^	SB^b^	DB^c^		
Surgery (min)	–	90 (±20)	105	53	NKLR and hospital financial records^d^
Material	–	1	2	400	
Brace	1	1	1	1063	Market price
Training/physio	52	52	52	44,6	NPTA^e^
Post-operative rehab (weeks)	–	12	12		

^a^ Nonoperative

^b^ Single bundle

^c^ Double bundle

^d^ Norwegian knee ligament register

^e^ Norwegian physiotherapist association

For the groups treated operatively, additional costs of surgery including allografts and extra fixation material for the DB alternative were added. As single-bundle procedure, we chose hamstring autograft with a commonly used fixation method as this has been most commonly used in the Scandinavian countries. The numbers for DB reconstruction were based on the technique described by Spiridonov et al. [19]. All operating expenses were calculated based on cost per minute and include surgeon and operating team fees, material used during surgery and administration costs (accounting costs from the authors’ institution). The price of the allografts was collected from standard price given by one of the major providers of allografts (Allosource^®^). For the SB and DB groups, the cost of post-operative rehabilitation following a standard programme of 12 weeks was also included. The cost of revision surgery has not been included as the numbers are small and because there are no separate numbers available for the DB group at present. The cost to society for absence from work has not been included in the analysis as these numbers are difficult to calculate within a population where a large proportion is students and/or athletes. The required time for a PCLR is taken from the average time used for isolated SB PCLRs reported to the Norwegian Knee Ligament Registry (NKLR) (112 procedures from 2004 to 2013) utilizing either hamstring or allograft. The registry holds no information on times for DB surgery. The use of NKLR data is approved by the national data inspectorate and the regional ethics committee. The additional time required for DB reconstruction was reported by two experienced PCL surgeons.

### Cost-effectiveness

The incremental cost-effectiveness ratio (ICER) is a statistic used in cost-effectiveness analysis to summarize the cost-effectiveness of a health care intervention. It is defined by the difference in cost between two possible interventions, divided by the difference in their health effect. It represents the average incremental cost associated with one additional unit of the measure of effect. The ICER can be estimated as: Incremental cost-effectiveness ratio (ICER) *=* (*C*_1_ − *C*_0_)/(*E*_1_ − *E*_0_), where *C*_1_ and *E*_1_ are the cost and effect in the intervention group and where *C*_0_ and *E*_0_ are the cost and effect in the control care group. Costs are usually described in monetary units, while effects can be measured in terms of health status or another outcome of interest. A common application of the ICER is in cost-utility analysis, in which case the ICER is synonymous with the cost per quality-adjusted life year (QALY) gained [2]. The cost of operative treatment was used to calculate the equivalent QALYs given a cost of €70,000 per QALY for both SB and DB surgeries. The expected increment in KOOS QoL was used to calculate a cost-effectiveness ratio (ICER) for SB reconstruction. Then, the same ICER value was used to estimate the required incremental gain in KOOS QoL for DB reconstruction.

## Results

The average calculated cost of nonoperative treatment was €3382. Incremental cost for SB PCLR was 154% and another 61% for DB PCLR, given that the preoperative rehabilitation programme is of the same length and intensity as that for the nonoperatively treated patients, details in Table 2. The additional cost of reconstruction on average equals the cost to society for 3 (SB)- or 6 (DB)-week absence from work in Norway. The allografts, material for extra fixation and time in surgery are the factors differentiating cost of the two surgical treatment options. In addition to the cost related to surgery, there is a cost of post-operative rehabilitation for both the SB and DB groups compared to that of the nonoperatively treated patients (Table 2). Given an expected gain in KOOS QoL of 27 points, this provides an incremental cost-effectiveness ratio (ICER) of 365. Adding the additional cost for DB reconstruction, this equals another relatively low incremental gain in QALYs. For a similar ICER score with DB over SB PCLR, this requires an incremental gain in KOOS QoL of 28 points (Table 3).

**Table 2:** Calculated cost of treating PCL injuries

Treatment/material	Nonoperative	Additional cost per surgery (Euro €)	
		Single bundle	Double bundle
Preoperative rehab	2319	2319	2319
Functional brace	1063	1063	1063
Surgery		3180	3975
Fixation material^a^		478	703
Allograft			4200
Post-op rehab		1545	1545
Total cost	3382	8585	13,805

^a^ Commonly used fixation devices used for both SB and DB reconstructions. Costs may vary according to supplier and institution

**Table 3:** Cost per quality of life measure (in €)

Intervention	Cost	Incremental cost	Incremental KOOS^d^	Required QALYs^e^	ICER–KOOS^f^
No^a^	3382				
SB^b^	8585	5203	27	0.074	365
DB^c^	13,805	5220	28	0.075	373

^a^ Nonoperative

^b^ Single bundle

^c^ Double bundle

^d^ Knee osteoarthritis outcome score

^e^ Quality-adjusted life year—threshold €70,000

^f^ Incremental cost efficiency ratio

## Discussion

The most important finding in the present study is that it can easily be argued that the gain in QoL-measures versus cost justifies the surgical option for the studied patient group. The average reported gain in KOOS QoL is about 27 points compared to no surgical treatment, which is a clinically relevant increase. The fact that the cost of PCLR is equivalent to a very small gain in QALYs supports this. The mentioned gain in KOOS may represent the ability of returning to work or sports at the same level as prior to injury, which in both cases may be of high value to the individual as well as to society.

Surgery adds a substantial extra cost in treating PCL injuries. Current literature reports good/excellent results following surgical reconstruction both when it comes to objective measures such as stress radiographs and when it comes to patient-reported outcomes. Taken into consideration that there is an additional cost related to DB surgery, one should demand a superior result for DB reconstruction that is both statistically significant and clinically relevant. For KOOS, the minimal clinically relevant change is about 10 points for the most relevant subscales [15]. If an additional gain in 10 points in KOOS QoL or Sport/Rec are enough to justify, the extra cost of DB surgery can be debated. For DB to be as cost-effective as SB, this would require an additional gain in KOOS QoL of 28 points which is a highly unlikely outcome.

When it comes to total cost, the differences between SB and DB reconstructions are relatively small when compared to the cost to society for work absence. If only one extra person per 100 treated would be able to return to work, or if those treated in general would be able to return to work more than 3 weeks earlier, DB surgery would have the same or lower cost as SB surgery. On average, we can estimate that patients have a sick leave period of 10 weeks following PCLR. Whether the required 3 weeks of earlier return to work is a realistic goal, is uncertain at best. A measure of the value of patients returning to sports is interesting, but difficult to calculate.

Sufficient data considering surgical revisions are not yet available to confirm whether the number of revisions is reduced after DB reconstruction. The current 5-year revision rate in Norway is about 4% which is similar to reported revision rates reported for ACL reconstructions in the Scandinavian countries [1, 9]. Even if the number of revisions were reduced to 0% following DB reconstruction, this alone would not be enough to save the extra money spent on switching form primary SB to DB reconstruction. Hence, a possible reduction in revision rate is not a sufficient argument for choosing DB over SB. This is based on the assumption that an average revision is done in one stage with the same post-operative rehabilitation programme used as for primary reconstructions.

The discrepancy in the reported operating time from the respective clinics can probably mainly be explained by the surgeons’ experience. Of the hospitals performing PCLR, eleven have reported only 1 PCLR per year. More experienced surgeons can probably reduce the duration of surgery even more, although the difference in time between single- and double-bundle surgeries should be fairly consistent as it only adds a relatively short standardized procedure. If this extra time should be considered, an extra cost is also a matter of discussion. The cost per minute in the OR is here calculated based on average numbers per minute from start in the morning until the day ends. It does not take into consideration how many patients are treated per day. Only 15 min extra time for the surgical procedure possibly implies that the same number of patients will be treated per day (8 am–4 pm in a typical clinic) for both SB and DB surgeries. This is because normally only one or two PCLRs are performed the same day and therefore combined with other relevant knee procedures in the relevant OR on the actual day. Then, we are left with the cost of the allografts and extra fixation material as the only additional cost for DB over SB.

With DB reconstruction, there is an increased volume of graft which theoretically transfers to more restrain to stress and improved stability. This is supported by biomechanical studies [20]. Some clinical studies report no difference in results or an advantage of DB reconstruction [14, 21]. For the high-performance athlete planning return to sport at preinjury level, DB reconstruction is a good treatment option as there are no studies implying inferior results with DB reconstruction. When it comes to other patient categories, both approaches should be considered. Although it can be argued that the extra cost with a DB technique is not so high that it should influence the method of choice given that one yields a superior result.

The cost to society and employers for time away from work following surgery comes in addition when compared to conservative treatment. If the PCL-injured patients are to be treated surgically with either method, one should expect both short-term and long-term results that justify this cost. There is of today no available literature comparing nonoperative treatment to operative treatment. There is one study available showing both statistically significant and clinically relevant improvement in KOOS scores 2 years following PCLR for an isolated PCL injury [11]. All these patients have gone through a period of rehabilitation prior to surgery. They have comparable increases in KOOS similar to that of patients treated for an anterior cruciate ligament injury. However, their KOOS scores are not even close to that of a control population with no prior knee injuries. The PCL patients ended up with a KOOS Sport/Rec score of 46 and a QoL score of 52 post-operatively. In a control population, the scores are 91 for Sport/Rec and 90 for QoL. It can be debated whether a clinically relevant increase in KOOS score is sufficient to justify operating on these patients or whether one should demand better outcomes. In addition, we do not yet know how good the results are after a longer period than 2 years. On the other hand, one could argue that the KOOS is not a linear scale and that an increase in the QoL subscale from 30 to 50 is an important increase as this translates to a better function in daily life activities and maybe a sufficient improvement for return to work for those that have moderately physical demanding work. This again translates to a benefit to society.

As there is room for improvement in post-operative results, one can argue that a central part of this is the surgical procedure. The only way to improve is to develop the reconstruction procedures and continue the research in the area.

A limitation to the data is the numbers representing total cost for surgical treatment. This is the cost in the authors’ hospital which should be comparable to other Norwegian and maybe even European hospitals, but there will be variations from country to country. These variations will also depend on how the costs are calculated. The cost to society will vary according to the organization of each country’s health services including who pays the hospital bill and how the expenses for work absence are calculated.

Another limitation may be the choice of procedure for DB surgery. This procedure has not been widely performed over years and not yet compared to other procedures for DB reconstruction. It is also an issue with allografts as they are not permitted for use in all countries. Another limitation is regarding the patients who are treated surgically without first going through a rehabilitation period of several months. For these patients who are treated more or less acutely, the first rehab cost is saved and leaves surgery as the only additional cost assuming the cost of preoperative and post-operative rehabilitation is equivalent. This economically favours early over later surgery. It could also be argued that twice a week with a physiotherapist is insufficient for an optimal rehabilitation. This has the potential of increasing the cost of preoperative and post-operative rehabilitation accordingly. QALY is a common parameter in evaluating the health outcome of new treatments. As data necessary to calculate QALYs are not available, this can be considered as a limitation to the current study. We will still argue that cost per gain in knee-related quality of life (KOOS QoL) is as good alternative measure to QALY for this patient group. Depending on which questionnaire or scoring tool that is applied for calculating QALY, one will get variation in the results. This again makes comparing studies looking at the same patient population less relevant if not the same questionnaire is for the QALY calculation.

In a clinical setting, the current study supports surgery as a treatment option in a health economic perspective as the cost is equivalent to a low gain in QALY. Both SB and DB reconstructions should be considered, keeping in mind that DB is less cost efficient than SB and as such has the potential of increasing cost with an uncertain incremental gain in QALY. In a future perspective, randomized controlled trials are needed to compare the outcomes of SB to DB PCLR including nonoperative treatment as a control group.

## Conclusion

There is a substantial extra cost to treating PCL injuries if it comes to surgical reconstruction. As the results following nonoperative treatment are reported to be good to excellent, it is difficult to justify the cost of surgery without first trying a nonoperative approach for isolated PCL injuries. If surgery is indicated, the method that most likely restores the biomechanics of the knee should be preferred almost irrespective of costs aspects.
